# When the novelty wears off: enemy spillover drives plant invasion success

**DOI:** 10.1111/nph.70486

**Published:** 2025-08-17

**Authors:** Ian A. Dickie, Warwick J. Allen, Lauren P. Waller

**Affiliations:** ^1^ School of Biological Sciences University of Canterbury Christchurch 8140 New Zealand; ^2^ Manaaki Whenua – Landcare Research Lincoln 7640 New Zealand; ^3^ Lincoln University Lincoln 7647 New Zealand

**Keywords:** apparent competition, enemy accumulation, herbivory, indirect interactions, invasion ecology, invasional meltdown, pathogens

## Abstract

Integrating plant physiological traits (ideal weed hypothesis) and interactions with enemies (enemy release hypothesis) may be key to understanding plant invasions. Contrary to enemy release, recent evidence suggests that invasive plants often accumulate generalist enemies due to the same r‐selected physiological traits that often drive invasive success. Despite high enemy loads, successful invasive plants can remain dominant due to high growth rates rather than due to lack of damage. Consequently, generalist herbivore and pathogen populations may be amplified by invaders and can spillover onto native plants, with these indirect interactions disproportionately affecting native rather than invasive plant species via apparent competition. Where this occurs, the generalist enemies of invasive plants may instead be hidden allies that amplify invader success and impacts.

**Table:** 

	Contents	
	Summary	1686
I.	Introduction	1686
II.	Enemy release is ephemeral	1687
III.	Invasive plants amplify enemy populations	1687
IV.	Enemies spillover onto native plants	1688
V.	An integrative framework with context dependency	1689
	Acknowledgements	1690
	References	1690

## I. Introduction

Plant invasions are both a major driver of ecosystem change and a great opportunity to understand the ecological processes involving range‐expanding species. Perhaps due to the importance of the subject, there has been a proliferation of hypotheses proposed to explain invasive species success (Gioria *et al*., 2023), many of which invoke either specific plant physiological traits (intrinsic drivers) or biological interactions (extrinsic drivers). For example, the ideal weed hypothesis suggests that invasive species have a particular set of physiological traits (e.g. self‐fertility, high dispersal, rapid growth rate, high specific leaf area and low reliance on obligate mutualists) that allow those species to achieve high biomass and rapid spread (Baker, 1965; Waller *et al*., 2020; Lau & Funk, 2023). Conversely, the enemy release hypothesis (Heger *et al*., 2024) along with some related concepts (e.g. enemy reduction, enemy‐of‐my‐enemy, enemy inversion, evolution of increased competitive ability and resource‐enemy release) all suggest that invasive plants should have fewer herbivores and pathogens or suffer less damage from those enemies than native plants and that this lower enemy load contributes to invasive success (and may allow increased allocation to growth over defence). Indeed, enemy release is a part of the theoretical basis behind the myriad biological control programmes around the world that seek to manage invasive plants by introducing their specialist enemies.

Here, we highlight a novel framework linking intrinsic and extrinsic drivers to understand how the physiological traits of the ideal weed hypothesis may drive plant–enemy interactions in invasion. Contrary to the idea of enemy release, recent evidence suggests that many invasive plants accumulate a high abundance of generalist enemies relative to native plants due to their physiological traits (e.g. high specific leaf area, aboveground biomass and/or population density, and growth rate). Despite high enemy loads, some invasive plants can achieve dominance due to high intrinsic growth rates rather than due to a lack of damage (Allen *et al*., 2021). A direct consequence of invasive plants tolerating high enemy loads through their high growth rates is the amplification of generalist herbivore and pathogen populations. Shared enemies can then spill over onto native plants, with these indirect interactions disproportionately affecting native rather than invasive plant species via apparent competition (Waller *et al*., 2024). We posit that where these indirect interactions occur, the generalist enemies of invasive plants may instead be hidden allies, and the accumulation of these enemies over time may amplify the success and impacts of invasive species.

## II. Enemy release is ephemeral

The enemy release hypothesis has been variously interpreted, but a recent summary of 38 prior reviews and meta‐analyses provides a revised definition of ‘A reduced pressure by enemies in the non‐native range contributes to invasion success’ (Heger *et al*., 2024). Enemy release fundamentally assumes some degree of host specificity of enemies and that enemies are dispersal‐limited (Brian & Catford, 2023). Despite being conceptually compelling and being supported for some species, there is numerically more evidence against the enemy escape hypothesis than supporting it, with better evidence for some specific component mechanisms than others (Heger & Jeschke, 2014). In particular, although invasive species often have lower abundance or diversity of pathogens in their non‐native than their native range, there is little evidence that they have fewer generalist pathogens, suffer less damage or have enhanced performance compared with natives (Heger & Jeschke, 2014; Brian & Catford, 2023).

The inconsistent support for enemy release may reflect temporal patterns. Meta‐analysis suggests that enemy release not only occurs after introduction but only lasts for as little as 50 yr (Hawkes, 2007). Following introduction, invasive plants accumulate enemies (Mitchell *et al*., 2010; Schultheis *et al*., 2015; Bertelsmeier *et al*., 2024) through the processes of co‐invasion (non‐native, co‐evolved organisms that are synchronously or asynchronously introduced) and adaptation or acclimation of resident enemies (native and introduced from other regions (Dickie *et al*., 2017)). This was originally called the pathogen accumulation hypothesis (Flory & Clay, 2013), but as parallel processes occur with herbivores, parasites and pathogens, it might be better termed enemy accumulation (Crous *et al*., 2017).

Enemy accumulation can occur through different mechanisms. For example, invasive plants that are phylogenetically related or share similar traits with natives may be prone to novel associations with semi‐generalist native herbivores and pathogens (Bufford *et al*., 2016; Crous *et al*., 2017). Co‐invasion, by contrast, may depend on the ability of enemies to arrive; therefore, it might be most common for invasive plants from geographically close or otherwise well‐connected locations, or for plant species that are frequently transported through international trade (Brian & Catford, 2023; Schertler *et al*., 2024). Co‐invading, novel and/or cosmopolitan generalist enemies are all likely to increase in populations once invaders spread and reach high abundance in their new range.

New incursions of invasive plants continue to occur at a constant rate, but the potential impacts of most plant invasions remain unrecognised until they have become well‐established. Most research on invasive plants has been conducted on long‐resident species for several reasons, such as their obvious environmental impacts, sufficiently widespread populations to provide replication for scientific study and fewer ethical constraints around leaving populations intact during the research. This suggests that, at least for widely recognised and heavily researched species, many invasives will have been present for sufficient time for enemy accumulation to have occurred.

## III. Invasive plants amplify enemy populations

Most enemies of invasive plants will be generalists, as observed in higher network connectivity of non‐native plant pathogens (Bufford *et al*., 2020), although a similar pattern is not necessarily apparent for invertebrate herbivores (Grandez‐Rios *et al*., 2015). Most successful invaders tend to be fast growing (Montesinos, 2022), due to both selection for species that rapidly colonise and dominate disturbed ecosystems, greater introduction effort of fast‐growing species (Gioria *et al*., 2023) and postintroduction evolution of rapid growth traits in the new range (Woods & Sultan, 2022). These fast‐growing, high‐biomass and highly fecund invasive plants may have low resistance to enemy damage, avoiding the costly constitutive defences needed to resist generalist pathogens and herbivores. Furthermore, it has been suggested that invasive plants may evolve to increase competitive traits (the evolution of increased competitive ability hypothesis), although evidence for this hypothesis has been mixed (Gioria *et al*., 2023).

High enemy loads on invasive plants have been frequently observed, including higher herbivore diversity, biomass and impacts on invasive compared with native plants in polyculture (Schultheis *et al*., 2015; Allen *et al*., 2021). However, high enemy loads do not necessarily reduce invasive success relative to natives. On the contrary, several studies have now shown that invasive plants are larger and more competitive in their new range despite harbouring large populations of enemies (Rotter & Holeski, 2018), especially generalists (Morrison & Hay, 2011; Waller *et al*., 2024). Maintaining high biomass despite high enemy loads may occur through very high intrinsic growth rates, such that loss of a high proportion of biomass occurs but does not alter competitive outcomes. Alternatively, invasive plants may have tolerance traits (e.g. photosynthetic compensation and resource reallocation) that can allow them to sustain high enemy populations without a major loss of biomass (Pagán & García‐Arenal, 2020) or higher fecundity (Woods & Sultan, 2022), allowing them to maintain higher enemy loads without reducing their population growth relative to native plants.

Soil feedback studies are challenging to interpret, as pathogen and root‐grazing herbivore loads are generally not quantified in these studies, and the measured outcome (plant growth) represents the integrated effects of abiotic and biotic drivers and plant physiological responses (De Long *et al*., 2023). Nonetheless, the observation of more negative plant–soil feedback on invasive compared with native plants in monoculture is not uncommon (Suding *et al*., 2013; Lustenhouwer *et al*., 2024; Waller *et al*., 2024; Chiarenza & Moles, 2025). Furthermore, a meta‐analysis of direct pairwise comparisons showed that home‐soil advantages are more common for native than for invasive species (Suding *et al*., 2013). Such feedbacks, when observed, are consistent with the possibility of invasive plants increasing populations in soil of pathogenic organisms and other antagonists that particularly negatively affect native species.

## IV. Enemies spillover onto native plants

Enemy spillover occurs where invasive plant populations increase populations of enemies that also affect co‐occurring native plants. This may include both non‐native and native enemies, sometimes distinguished as spillover and spillback, respectively (Dickie *et al*., 2017; Bard *et al*., 2024), but here we use ‘spillover’ as the inclusive term for both. Ecosystems dominated by non‐native plants have been shown to support a high diversity and abundance of insect herbivores (Bertelsmeier *et al*., 2024) and fungal parasites and pathogens (Makiola *et al*., 2019) and support *Phytophthora* that impact native plants (Lewis *et al*., 2019). Selected examples of sharing of potential enemies between non‐native and native plants have been observed in the invasion of myrtle rust in Hawaii (Dickie *et al*., 2017), fungal communities in invaded grasslands (Visscher *et al*., 2021), foliar endophytes and pathogens of herbaceous weeds (Chen *et al*., 2020) and herbivore assemblages of native and non‐native lineages of *Phragmites australis* in North America (Cronin *et al*., 2015).

**Fig. 1:**
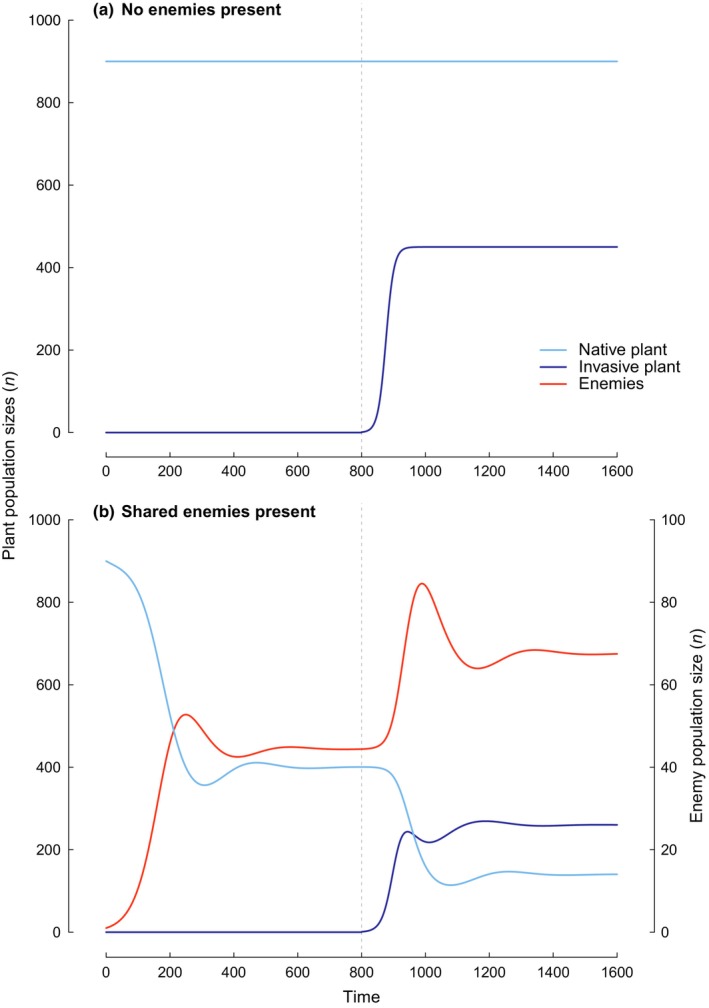
Lotka–Volterra models of two noninteracting plant populations (native and invasive), with the invasive species arriving at time point 800 (grey vertical dashed lines). The native plant is assumed to have a high carrying capacity (*K*) and low intrinsic population growth rate (*r*) relative to the invasive plant. In the absence of enemies (a), both species co‐exist at their respective *K* values given the simplifying assumption of no niche overlap. In the presence of enemies (b), the native plant co‐exists with enemies before the arrival of the invasive plant (Time < 800) but is rapidly reduced in local population size (or density) following the arrival of the invasive plant due to the increase in enemy populations and the higher r value of the invasive plant. Units are arbitrary. An interactive version of the model allowing exploration of parameter space (including with competition) is available in R by first installing the packages deSolve and shiny and then running: ‘runGitHub (“EcosystemMycologist/EnemySpillover”)’. Code modified from a modification of Stevens (2009) by D. B. Stouffer (pers. comm.).

Notwithstanding the complexities of plant–enemy interaction networks in invasion, a simple Lotka–Volterra model demonstrates that few assumptions are required for enemy spillover to drive large reductions in native plant populations (Fig. 1). Specifically, where invasive plants have a higher intrinsic growth rate than co‐occurring native plants, and they share enemies, the population of native plants is greatly reduced. This occurs even where the native plant has a higher carrying capacity and would be capable of co‐existing with enemies in the absence of invasive plants or with invasive plants in the absence of enemies. It also occurs even where the invasive and native plants do not otherwise compete.

Outside a simplified modelling framework, the net effect of enemy spillover in a recipient community will depend on how non‐natives are affected relative to native plants. Accumulated enemies may increase invader establishment and spread if enemies accumulated by high‐density non‐natives spillover onto natives and have stronger negative effects on the natives compared with the non‐native plants (Flory & Clay, 2013; Waller *et al*., 2024), known as apparent competition (Holt, 1977). For example, accumulated enemies may disadvantage non‐native plants growing in monoculture, but when grown in competition with native plants, the net effect of enemies may be neutral or even positive on non‐natives (Waller *et al*., 2024).

## V. An integrative framework with context dependency

**Fig. 2:**
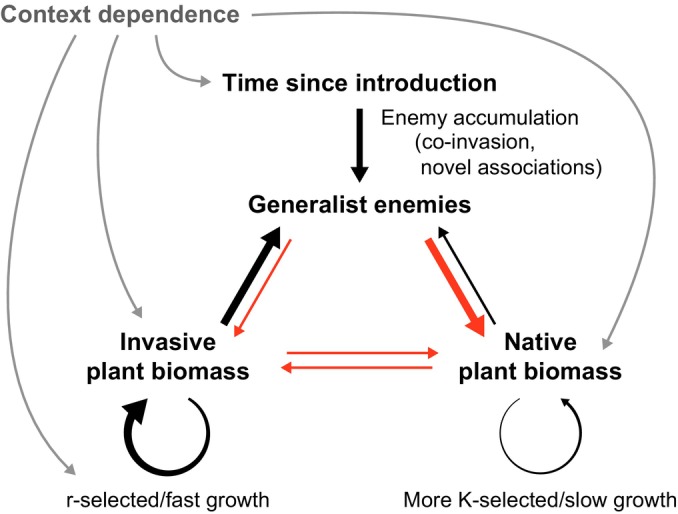
Schematic model of the enemy spillover framework showing positive (black) and negative (red) feedbacks, along with context dependence. The framework proposes that the intrinsic fast‐growth traits of many successful invasive plants lead to strong positive effects on generalist enemies, which then impact relatively more strongly on more K‐selected native plants. Direct negative interactions (competition) are shown but are not necessary for indirect interactions via shared enemies to drive invasive success. Thicker arrows reflect stronger effects.

Plant invasion ecology has been criticised for a proliferation of hypotheses, often in the absence of empirical data (Gioria *et al*., 2023). Here, we are not proposing a new hypothesis for understanding plant invasion but rather showing how several existing hypotheses combine to provide a more holistic understanding of invasion dynamics (Fig. 2). In particular, our framework demonstrates the importance of intrinsic factors (the ideal weed hypothesis) as drivers of extrinsic factors (enemy accumulation and spillover). Although the accumulation of enemies on invasive plants has often previously been considered a factor that might limit invasions (e.g. Dickie *et al*., 2017), our framework posits that enemy accumulation on invasive plants with fast growth traits is actually exacerbating invasive impacts on plant communities.

Brian & Catford (2023) identified seven important contexts for the enemy release hypothesis (Table 1). Although their conceptual review did not consider interactions with the broader community (i.e. indirect interactions/spillover/apparent competition), all seven contexts (and two additional) could also influence the strength of indirect effects mediated by shared generalist enemies (Table 1).

**Table 1:** Many of the context dependencies identified by Brian & Catford (2023) for the enemy release hypothesis also apply to our framework. In addition to the seven dependencies identified by Brian and Catford (2023), we add biomass and biogeography.

Context dependency	Application to framework
Time since introduction	Indirect effects will become stronger with time since introduction due to population growth of the invader, accumulation of enemies and (possibly) the evolutionary loss of plant defences.
Resource availability	Invaders from a native range with high resources will exert stronger indirect effects due to their low defences, fast growth rates and therefore rapid accumulation of enemies. High resources in the invaded range may facilitate investment in either (or both) growth or defence.
Phylogenetic relatedness of invasive and native species	Indirect interactions will be stronger with higher functional and/or phylogenetic relatedness of invasive and native species (Grandez‐Rios *et al*., 2015; Bufford *et al*., 2016).
Host–enemy (–host) asynchronicity	Indirect effects will be weaker with increasing host–enemy (–host) asynchronicity in space or time.
Number of introduction events	The number of introduction events could influence indirect interactions in a variety of ways, depending on whether co‐introduced enemies are specialists (reduced enemy release and apparent competition) or generalists (reduced enemy release but potentially increased apparent competition; e.g. co‐introduced herbivores of *Phragmites australis* in North America (Bhattarai *et al*., 2017)).
Type of enemy	The strength of indirect interactions will depend on the type of enemy. For example, spillover will be more likely to occur with generalist than with specialist enemies, but indirect effects may be stronger when mediated by enemies with narrower host ranges (Taylor & Snyder, 2021), compared with more diffuse apparent competition mediated by ‘true’ generalists. Brian & Catford (2023) also mention other enemy traits such as taxonomic/functional group, size and feeding guild.
Growth‐defence trade‐offs	The ability to exert indirect interactions will be stronger for invasive species with a strong growth‐defence trade‐off (i.e. high growth and low defences).
Biomass	Enemy spillover requires the invasive species to reach sufficient biomass relative to native species to drive an increase in enemy populations.
Biogeography	The strength of enemy release and indirect interactions can vary spatially, such as along latitudinal gradients (Cronin *et al*., 2015; Bhattarai *et al*., 2017).

Perhaps the most important context for indirect interactions is that plants need to be considered in communities rather than in monocultures. For example, negative plant–soil feedbacks appear stronger on invasive plants grown in monocultures than in communities (Waller *et al*., 2024) and shared enemies with natives will likely reduce invasive growth in communities (Shan & Hou, 2024). Nonetheless, enemy spillover can drive a reversal of the net outcome when negative enemy impacts are lower for invasives relative to the natives (Waller *et al*., 2024). Thus, far from escaping their enemies, invasive plants with fast growth rates can aid and abet the impact of these enemies on native ecosystems.

It is unlikely that a single hypothesis can explain the success of all invaders. Whether an invasive plant benefits from novel enemy interactions (or escape from those interactions) may come down to the traits of invaders (Leishman *et al*., 2007), herbivore feeding styles, pathogen life‐history traits and evolutionary relationships (Bufford *et al*., 2016). Low‐biomass and slower growing invasive plants may have other important impacts but are unlikely to drive enemy spillover, as low‐biomass populations are unlikely to support substantially increased generalist enemy populations. Impacts on native plants will also be contingent on their species‐specific traits. Notwithstanding these limitations, our framework has a high probability of applying to most well‐established, widespread and high‐biomass invasive species. As such, we suggest that further research that integrates intrinsic and extrinsic drivers of invasive success across a range of community and environmental contexts is needed to better understand the most problematic and widespread plant invasions.

## Competing interests

None declared.

## Author contributions

All three authors (IAD, WJA, LPW) contributed to developing the ideas, writing and editing this manuscript. IAD wrote the model in Fig. 2. IAD, WJA, LPW contributed equally to this work.

## Disclaimer

The New Phytologist Foundation remains neutral with regard to jurisdictional claims in maps and in any institutional affiliations.
